# Abnormalities in Glucose Metabolism, Appetite-Related Peptide Release, and Pro-**i**nflammatory Cytokines Play a Central Role in Appetite Disorders in Peritoneal Dialysis

**DOI:** 10.3389/fphys.2019.00630

**Published:** 2019-05-28

**Authors:** Lorena Avila-Carrasco, Mario A. Pavone, Elena González, Álvaro Aguilera-Baca, Rafael Selgas, Gloria del Peso, Secundino Cigarran, Manuel López-Cabrera, Abelardo Aguilera

**Affiliations:** ^1^Unidad Académica de Medicina Humana y Ciencias de la Salud, Universidad Autónoma de Zacatecas, Zacatecas, Mexico; ^2^Servicio de Nefrología Hospital Can Misses, Ibiza, Spain; ^3^Servicio de Nefrología, Instituto de Investigación Biomédica Princesa, Hospital Universitario la Paz, Universidad Autónoma de Madrid, Madrid, Spain; ^4^Facultad de Ciencias Médicas, Hospital Escuela, Universidad Nacional Autónoma de Honduras, Honduras, Honduras; ^5^Servicio de Nefrología, Hospital da Costa–Burela, Burela, Spain; ^6^Centro de Biología Molecular-Severo Ochoa, Consejo Superior de Investigaciones Científicas–Universidad Autónoma de Madrid, Madrid, Spain

**Keywords:** appetite peptides, pro-inflammatory cytokines, insulin resistance, fat tissue gene expression, peritoneal dialysis, euglycemic–hyperglycemic clamp

## Abstract

**Background:** Appetite disorders are frequent and scantly studied in peritoneal dialysis (PD) patients and are associated with malnutrition and cardiovascular complications.

**Objective:** We investigated the relationship between uremic insulin resistance, pro-inflammatory cytokines, and appetite-related peptides release (ARPr) with eating-behavior disorders in PD patients.

**Methods:** We included 42 PD patients (12 suffering anorexia, 12 obese with high food-intake, and 18 asymptomatic) and 10 controls. We measured blood levels of ARPr including orexigens [neuropeptide-Y (NPY), ghrelin, and nitric-oxide], anorexigens [cholecystokinin, insulin, corticotropin-releasing factor, leptin, and adiponectin (Ad)], and cytokines (TNF-α, sTNFα-R2, and IL-6) both at baseline and after administering a standard-food stimulus (SFS). We also measured the expression of TNF-α, leptin and Ad-encoding mRNAs in abdominal adipose tissue. We compared these markers with eating motivation measured by a Visual Analog Scale (VAS).

**Results:** Anorexics showed both little appetite, measured by a VAS, and low levels of orexigens that remained constant after SFS, coupled with high levels of anorexigens at baseline and after SFS. Obeses showed higher appetite, increased baseline levels of orexigens, lower baseline levels of anorexigens and cytokines and two peaks of NPY after SFS. The different patterns of ARPr and cytokines pointed to a close relationship with uremic insulin resistance. In fact, the euglycemic–hyperglycemic clamp reproduced these disorders. In anorexics, TNF-α fat expression was increased. In obese patients, leptin expression in fat tissue was down-regulated and showed correlation with the appetite.

**Conclusion:** In PD, appetite is governed by substances that are altered at baseline and abnormally released. Such modulators are controlled by insulin metabolism and cytokines and, while anorexics display inflammatory predominance, obese patients predominantly display insulin resistance.

## Introduction

Malnutrition is a severe and frequent (20–40%) complication in peritoneal dialysis (PD) patients. Its cause is not entirely known but it has been associated with the accumulation of pro-inflammatory cytokines, as well as metabolic and eating behavior disorders (EBD) (Owen et al., 1999). EBD in PD patients may range from anorexia to obesity with high food intake. Anorexia is the main obstacle to achieve an adequate nutritional status, while obesity is frequently associated with protein malnutrition, inducing a Kwashiorkor-like syndrome (Aguilera et al., 2004b).

In renal patients there is an accumulation in plasma of uremic toxins., PD is an essential daily life-saving treatment for end-stage renal failure and involves the exchange of solutes and the excess of water between blood and dialysis solution across the peritoneal membrane, which results in gradual reduction of uremic solutes and toxins (Raby and Labéta, 2018; Nigam and Bush, 2019). Reduced appetite (anorexia) is an early and usual symptom of uremic syndrome (Carrero et al., 2007, 2008). Anorexia in dialysis patients has been historically considered as a sign of uremia due to “inadequate” dialysis. Additionally the inflammation may also play a role in the genesis of appetite disorders in these patients (Aguilera et al., 2004b; Chazot, 2009).

Appetite is regulated by numerous organs, including the liver, the gastrointestinal tract (GIT) and the brain. The hypothalamus, is the main regulating region of the brain for appetite and energy homeostasis, involving counter-regulation of appetite by orexigenic molecules, including neuropeptide-Y (NPY), ghrelin and nitric-oxide and anorexigenic molecules, such as proopiomelanocortin (POMC), cholecystokinin (CCK), insulin, corticotropin-releasing factor (CRF), leptin, adiponectin (Ad), and pro-inflammatory cytokines (Yu and Kim, 2012).

It has been shown that low NPY serum levels are associated with anorexia in PD patients (Aguilera et al., 1998, 2004a; Yu and Kim, 2012). NPY is a 36-amino acid peptide and is the most abundant and widely distributed neuropeptide in the human brain (Adrian et al., 1983). In the periphery, NPY is expressed primarily in sympathetic ganglia, the adrenal medulla, and in platelets (Larhammar, 2001; Hirsch and Zukowska, 2012). The main effects of NPY are increased food intake and decreased physical activity. It also increases the proportion of energy stored as fat and blocks nociceptive signals to the brain. In regards of its role in obesity, it has been shown that an increase in NPY is caused by high levels of glucocorticosteroids through directly activating type II glucocorticosteroids receptors and indirectly, by abolishing the negative feedback of CRF on NPY synthesis and release (Brothers and Wahlestedt, 2010).

Corticotropin-releasing factor is a 41-aa neuropeptide secreted by neurons in the paraventricular nucleus of the hypothalamus (Vale et al., 1981), this has a role in the regulation of ACTH release and also the major physiological regulator of the hypothalamic–pituitary–adrenal (HPA) axis (Smagin et al., 2001; Sabzevari et al., 2019), and has an important role in the control of food intake (Tanaka et al., 2009). CRF has been measured in peripheral plasma in several diseases, stress situations, and HPA disorders and it has been suggested that the major proportion of plasma CRF has a hypothalamic origin (Suda et al., 1985; Cunnah et al., 1987; Ellis et al., 1990; Wittert et al., 1991; Donald et al., 1994; Catalán et al., 1998).

The pathogenesis of the wide spectrum of appetite disorders in uremia is unknown. The accumulation of appetite-related peptides (ARPs) and pro-inflammatory cytokines (Aguilera et al., 1993) upon renal failure may alter the hunger-satiety cycle. Both ARPs and cytokines might interact and potentiating each other, producing a wide range of eating behavior responses. In this context, it has been shown that CCK and interleukin-1 (IL-1) may synergize to worsen anorexia and that TNF-α may interfere with the orexigenic effect of neuropeptide-Y (NPY) (Xu et al., 1988; Daun and McCarthy, 1993).

Given the proposed definition of “malnutrition, inflammation, and atherosclerosis” (MIA) syndrome, dialysis patients may suffer from two types of malnutrition: Type-I malnutrition, in reference to a severe form of cachexia due to high plasma levels of inflammatory mediators that produce systemic catabolic effects, being anorexia one of the most important symptoms; and Type-II malnutrition, that is usually mild and reversible, and that is barely mediated by inflammation (Stenvinkel et al., 2000).

The other important groups of features that regulate appetite are associated with glucose and insulin metabolism (Isganaitis and Lustig, 2005). It is well known that uremic state is associated with insulin resistance (Zoccali et al., 2005) and a clear relationship between insulin release, glucose metabolism, ARP-release (ARPr) and pro-inflammatory cytokines has been established (Kalra et al., 2008). IL-1 and TNF-α induce insulin resistance and the sense of satiety that acts on the hypothalamus (central anorexia) (Isganaitis and Lustig, 2005). Some ARP and cytokines retained during renal failure contribute to the perpetuation of insulin resistance and to the establishment of chronic appetite disorders (Aguilera et al., 2004a; Zoccali et al., 2005). In this sense, fat tissue plays a crucial role in insulin metabolism, controlling the release of peptides and cytokines (Isganaitis and Lustig, 2005; Axelsson, 2008). The abnormalities in leptin, Ad or resistin fat gene expression indicate that the influence of fat tissue on such metabolism is particularly affected by uremia (Kalra et al., 2008).

We were interested in defining the relationship between ARPr, circulating cytokines, insulin resistance, fat gene expression and EBD in PD patients. This population is of particular interest, since PD patients are exposed to constant peritoneal glucose absorption, which may worsen disorders of glucose metabolism and directly induce central anorexia (Aguilera et al., 2004b; Kalra et al., 2008). In some cases, the tendency toward high plasma levels of anorexigen peptides is thought to reflect the disequilibrium between orexigens and anorexigens (Aguilera et al., 2004b). However, not all PD patients suffer anorexia under similar conditions, suggesting that factors like peritoneal glucose absorption and insulin metabolism, or dialysis clearance, influence on appetite control. Accordingly, we hypothesize that disturbances in the secretion and/or renal retention of ARP and inflammatory cytokines, all of which act on fat tissue and are associated with insulin resistance, induce different EBD, ranging from anorexia to obesity with high food intake.

## Materials and Methods

We included 42 clinically stable PD patients (20 males and 22 females), 20 with continuous ambulatory peritoneal dialysis (CAPD) and 22 with automated PD. The causes of chronic renal failure were nephrosclerosis in 15 patients, glomerulonephritis in 11, polycystic kidney disease in 8, systemic disease in 4 and unknown in 4. Of these 42 PD patients: 12 suffered anorexia, 12 were obese with high food-intake and 18 were asymptomatic. We excluded diabetic patients, and those suffering from neoplasias, chronic or acute infections, liver, and rheumatoid diseases. We also included 10 healthy controls. This research was carried out in accordance with Good Clinical Practice Guidelines, applicable regulations as well as the ethical principles that have their origin in the Declaration of Helsinki. All of included patients signed a written consent.

### Dialysis Adequacy and Nutritional Markers

Urea-Kt/V and nPNA (normalized protein nitrogen appearance) were calculated by standard methods (Selgas et al., 1993). Long-term nutritional markers in plasma/serum were analyzed: creatinine, albumin, cholesterol (colorimetric method, Hitachi 704) and transferrin were assayed by immunonephelometric methods (Boering Nephelometric-Terminal S.A., Spain) and serum iron (Hitachi 911). Medium-term nutritional markers were also assessed, plasma prealbumin and retinol-binding protein (RBP) immunonephelometric method). Serum growth hormone (GH) was determined by ELISA (AIA 1200; Tosoh Corporation, Tokyo, Japan), normal value <5 ng/mL. Serum IGF-I was determined by radioimmunoassay, RIA(Nichols Institute Diagnostics, San Juan Capistrano, CA, United States), normal range between 83 and 450 ng/mL. The short-term nutritional markers analyzed in serum were: urea nitrogen, phosphate, and potassium. The mean daily dietary intake was determined from the individual 24-h food records over a 3-day period (Food composition tables, Wander-Sandoz Nutrition, Barcelona, Spain; Jiménez Cruz et al., 1990).

Anthropometric parameters explored were: triceps skin-fold (TSF), mid-arm circumference (MAC), and mid-arm muscle circumference [MAMC (cm) = MAC (cm) – 3.14 × TSF (cm)]. The TSF was determined using a caliper (Holtain Ltd., Cross-well, Crymych, Dyfed, United Kingdom). Body composition was determined through bioelectric impedance (BI: multi-frequency, Maltron BF 905, United States).

### Eating Motivation Analysis

To evaluate eating motivation in the patients, we use Visual Analog Scale (VAS), which includes five questions. The results were given in a horizontal scale (0–100) (Hylander et al., 1997). Anorexia was defined by three criteria: low eating motivation measured by VAS, that was considered when the mean of all answers was <60; low food intake (nPNA < 1.1 g/kg/day and daily dietary assessment <35 kcal/kg/day); and low nutritional markers (according to KDOQI clinical practice guideline of nutrition) (Kopple, 2001; Aguilera et al., 2004b).

Obesity with high food intake was considered when the BMI was higher than 30 kg/m^2^, and eating motivation (VAS) and daily food intake was high (Hylander et al., 1997).

According to body composition, nutritional status, eating motivation and the knowledge derived from eating behavior disorders studied in other medical areas (Robins et al., 1988; American Psychiatric Association, 2013), we divided our patients into three groups: suffering anorexia (*n* = 12), obesity (*n* = 12) with high food intake or without EBD (*n* = 18). Finally, we included a control group of 10 health volunteers.

We evaluate the appetite peptide modulators at the baseline (fasting condition), as well as 30, 60, and 90 min after the ingestion of a standard 750 mL nutritional supplement (Fresubin^TM^, Fresenius, Medical Care, Germany). They were analyzed according to manufacturer recommendation. These peptides included:

### Hormones and Peptides Related to Insulin Resistance

(1) Glucose: assayed by the hexokinase reaction (Boehringer Mannheim, Germany). The normal fasting range from 90 to 120 mg/dL.
(2) Insulin (Sorin; Biomedica, Saluggia, Italy): normal range 10–15 μU/mL.
(3) C-peptide (Medigenix; Diagnostics Fleurus, Belgium): normal range 0.5–3 ng/mL.
(4) Glucagon (ICN Biomedicals, Irvine, CA, United States): normal range 70–90 pg/mL.
(5) Gastric inhibitory peptide (GIP; ELISA, Peninsula Laboratories, Inc., Belmont, CA, United States): normal range 35–52 pg/mL.

### Insulin Resistance (IR) Test

(1) Insulin resistance (IR) was estimated using the homeostatic model assessment (HOMA-IR). HOMA-IR = Fasting insulin (μU/mL) × fasting glucose (mg/dL)/405. Low-IR values indicate high insulin sensitivity, whereas high-HOMA values indicate low insulin sensitivity (insulin resistance) (Matthews et al., 1985).
(2) Euglycemic hyperinsulinemic clamp studies were performed to discriminate the effect of hyperglycemia and hyperinsulinemia separately on ARPr. We followed the methodology of DeFronzo et al. (1979).

#### Anorexigenic Peptides

(1) Cholecystokinin, the 26–33 unsulfated fragment (ELISA, Peninsula Laboratories, Inc., Belmont, CA, United States): normal values 12–20 pg/mL.
(2) Leptin (RIA, Linco Research, St. Louis, MO, United States): normal range 3–7.8 ng/mL.
(3) Adiponectin (Ad; RIA, Linco Research, St. Charles, MO, United States): normal values in our population were 25–33 μg/mL.
(4) Corticotropin-releasing factor (CRF; ELISA, Easia Medigenix Diagnostics S.A., Belgium): normal range 20–40 pg/mL.

#### Orexigenic Peptides

(1) Neuropeptide Y (NPY; ELISA, Peninsula Laboratories, Inc., Belmont, CA, United States): normal range 220–370 pg/mL.
(2) Nitric oxide (NO) measured as serum nitrate NO_3_, a final metabolite of NO, by capillary electrophoresis: normal range in 109 healthy volunteers was between 90 and 110 μmol/L.
(3) Ghrelin, the ^125^I-Ghrelin (RIA, Linco Research, St. Charles, MO, United States): normal range 900–2,500 pg/mL.

#### Cytokines Acting on Appetite Control

(1) Tumor necrosis factor (TNF-α) and IL-1, were determined by ELISA (Easia Medigenix, Diagnostics S.A., Belgium), normal ranges were 3–20 pg/mL and 1–16 pg/mL, respectively.
(2) IL-6 (R&D Systems, Minneapolis, MN, United States: normal range 0.25–1.5 pg/mL).
(3) TNF-α receptor-2 (sTNFα-R2; BioSource Europe, Nivelles, Belgium: normal range 1–7.8 ng/mL).

### Quantitative RT-PCR Analysis in Abdominal Fat Tissue

Gene expression was assayed in abdominal subcutaneous adipose tissue obtained from elective surgery (controls) and PD catheter placement when patients started PD or during PD catheter exchange. The samples were placed into warm saline, snaps frozen in liquid nitrogen and stored at -80°C. For quantitative RT-PCR analysis frozen tissue samples were lysed in TRI-Reagent (Ambion, Inc., Austin, TX, United States) using a Polytron homogenizer to extract total RNA. Complementary DNA was obtained from 1 μg of RNA by reverse transcription according to manufacturer’s recommendations using High Capacity RNA to cDNA kit (Applied Biosystems, Cheshire, United Kingdom) as described previously (Sandoval et al., 2016). Quantitative PCR was carried out in a Light Cycler 480 using the FastStart Universal Probe Master (ROX) (Roche Applied Science) with the following program: 50°C, 2 min; 95°C, 10 min; 60 cycles of 95°C, 15 s and 60°C, 10 s; 4°C; and specific primers sets for human adiponectin, leptin, TNF-α and glyceraldehyde-3 phosphate dehydrogenase (GAPDH) mRNAs (Supplementary Table S1) (Ruiz-Carpio et al., 2017). Data normalized to the housekeeping gene TBP were analyzed using the 2^-ΔΔCt^ method.

### Statistical Analysis

Results are given as the mean ± SD and range. The comparisons between groups were performed using a non-parametric test, the Mann–Whitney rank-sum *U* test. Spearman regression analysis and “*t*” student tests were used for paired and non-paired data. To analyze the statistical differences between the groups, the ARPr serum curves and the VAS (tables and graphic curves) we used the variance multi-factor analysis (ANOVA). Figure 4, box plots show the 25th and 75th percentiles, median, minimum and maximum values of five independent experiments. The symbols represent the statistical differences between the groups. In figures, statistic differences between the groups data were performed using triple factor ANOVA test and Mann–Whitney rank sum *U* test using the SPSS statistic package version 14.5 (Chicago, IL, United States) and GraphPad Prism version 4.0 (La Jolla, CA, United States). A “*p*” value less than 0.05 was considered statistically significant.

## Results

The demographic and basal biochemical characteristics of the subjects are shown in Table 1. The most relevant findings were that anorexic patients were older and had significantly lower levels of nutritional markers and residual renal function (creatinine clearance). No differences were found in the frequency of EBD between CAPD vs. automated techniques. In CAPD group, seven from twelve patients were anorexics, six obese and eight were asymptomatics. Similarly, there were no differences in the distribution of EBD by gender. Six from 12 women were anorexics, seven obese, and nine were asymptomatics.

**Table 1:** Baseline differences between the groups (PD patients).

Parameter	Anorexic	Obese	Asymptomatic	Controls	*P*
Age (years)	66.4 ± 10(a,d)	56.3 ± 7.1(b)	49.7 ± 14(a,c)	43 ± 4.7(b,d)	(a,b,c,d) < 0.05
DP duration (m)	36.8 ± 32.3	23 ± 11.5	45.5 ± 46.7		NS
CCr (mL/min)	0.5 ± 0.45(a)	1.42 ± 1.01(b)	1.38 ± 1.39(c)	101 ± 7(a,b,c)	(a,b,c) < 0.00
nPNA (g/kg/día)	0.87 ± 0.21(a)	1.1 ± 0.25	1.14 ± 0.11(a)		(a) < 0.05
KT/V de urea	2 ± 0.25	1.98 ± 0.33	2.17 ± 0.33		NS
Serum Urea (mg/dL)	152 ± 21(a)	159 ± 45(b)	146 ± 51(c)	28 ± 4(a,b,c)	(a,b,c) < 0.00
Cr (mg/dL)	10.4 ± 2(a)	11.3 ± 2(b)	10.5 ± 3(c)	1 ± 0.2(a,b,c)	(a,b,c) < 0.00
Cholesterol (mg/dL)	174 ± 57.4	211 ± 55.6	188 ± 56	184 ± 30	NS
Albumin (g/dL)	3.7 ± 0.08(a,b)	4 ± 0.2(a)	3.9 ± 0.4	5 ± 0.4(b)	(a,b) < 0.05
Transferrin (mg/dL)	209 ± 36	262 ± 47	205 ± 50.7	303 ± 57.2	NS
Prealbumin (mg/dL)	26 ± 7(a,b)	31 ± 2.9(a)	31 ± 7.5	34 ± 3(b)	(a,b) < 0.05
RBP (mg/dL)	8.4 ± 3(a,b)	11.5 ± 3(a)	13 ± 2(b)	5.3 ± 1.2	(a,b) < 0.05
IGF-I (ng/mL)	257.5 ± 122(a)	370 ± 142.7(a)	365 ± 224.6	205 ± 91.2	(a) < 0.05
GH (ng/mL)	3.4 ± 3.8	4 ± 4.8	2.2 ± 1.4	1.7 ± 1.7	NS
TSF (cm)	9.5 ± 4(a,b)	24.2 ± 11.4(a)	22.3 ± 9.4(b)	19.9 ± 10.1	(a,b) < 0.05
BSF (cm)	4 ± 0.6(a,b,c)	18.6 ± 7.3(a)	11.4 ± 8.6(b)	13.7 ± 9.5(c)	(a,b,c) < 0.05
AMMC (cm)	24.2 ± 1.4(a,b)	27.4 ± 2(a,c)	23.9 ± 13(c)	36.1 ± 13(b)	(a,b,c) < 0.05
Diet survey (kcal/day)	1277 ± 467.4(a,b,c)	2320 ± 179.4(a)	2006 ± 351(b)	2089 ± 339(c)	(a) < 0.01 (b,c) < 0.05
Fat (kcal/day)	60.4 ± 28.9(a,b)	102 ± 23.2(a,c)	98 ± 22(b)	74.7 ± 15(c)	(a,b,c) < 0.05
Proteins (kcal/day)	63 ± 18(a)	85.7 ± 16.6(a)	83.8 ± 13.7	74.5 ± 21.8	(a) < 0.05
Carbohydrate (kcal/day)	98 ± 41(a,b,c)	227 ± 71(a)	155.5 ± 27(b)	248.8 ± 67(c)	(a,b,c) < 0.01
BIP. BMI (kg/m^2^)	23 ± 2(a)	31.1 ± 3(a,b)	25 ± 2	24 ± 1.5(b)	(a,b) < 0.05
BIP. Lean (kg)	22.8 ± 3.7(a,b)	30.4 ± 4.5(a)	25.4 ± 2.8	28.6 ± 5.7(b)	(a,b) < 0.05
BIP. Fat (kg)	18 ± 5.4(a)	25 ± 8.8(a,b)	13.5 ± 5.3(b)	18.7 ± 4.2	(a,b) < 0.05
IPB. Water (L)	35.7 ± 5.3(a)	43.7 ± 7.7(a)	37.7 ± 5.4	36.6 ± 5.8	(a,b) < 0.05
TNF-α (pg/mL)	121 ± 43.8(a,b,c)	40 ± 11.6(a,c)	38.2 ± 16(b,c)	16 ± 4(c)	(a,b) < 0.01 (c) < 0.001
IL-1 (pg/mL)	6.12 ± 0.8(a,b,c)	2.1 ± 0.43(a,c)	2.2 ± 1.34(b,c)	1 ± 0.8(c)	(a,b,c) < 0.001
IL-6 (pg/mL)	9.3 ± 3(a,b)	11 ± 5.2(c,d)	5.4 ± 2(a,d)	2 ± 1.2(b,c)	(a,b,c) < 0.05 (c) < 0.01
Adiponectin (pg/mL)	29.2 ± 4.2(a)	20.1 ± 7.8(b)	30.3 ± 7.7(c)	9.8 ± 2.4(a,b,c)	(a,b,c) < 0.01
HOMA-IR	7.8 ± 1.9(a,b)	10.4 ± 5.1(c,d)	3.5 ± 0.8(a,c)	2.48 ± 0.04(b,c,d)	(a) < 0.05 (b,c,d) < 0.01

*Letters represent the statistical differences between the groups (read in horizontal). CCr, creatinine clearance. Cr, serum creatinine. RBP, retinol protein binding. IGF-I, insulin growth factor type I. GH, growth hormone. TSF, tricipital skin fold. BSF, bicipital skin fold. AMMC, arm muscular mean circumference. BIP, bioelectric impedance. BMI, body mass index. (a–d) Represents the statistical differences between the groups. Statistical comparisons were made group by group (Mann–Whitney U test).*

### Visual Analog Scale (VAS)

The analysis of the VAS applied to the different groups indicated that the anorexic patients had a lower eating motivation and obese the higher eating desire (Table 2).

**Table 2:** Eating motivation measured with VAS in PD patients suffering EBD.

VAS	Patients	Anorexics (*n* = 12)	Obese (*n* = 12)	Asymptomatics (*n* = 18)	Controls (*n* = 10)	*P*
Desire to eat before lunch		60 ± 6.1 (a,b)	76.6 ± 6 (a)	67.8 ± 6.9	72.8 ± 3.9 (b)	(a,b) < 0.01
Desire to eat after lunch		8.6 ± 2.2 (a)	21.6 ± 4 (a)	13.2 ± 5	13.5 ± 8.5	(a) < 0.05
Hunger before lunch		60 ± 6.1 (a,b,c)	78.3 ± 6 (a)	68.6 ± 4.7 (b)	74.3 ± 4.5 (c)	(a) < 0.01 (b,c) < 0.05
Hunger after lunch		8 ± 4.4 (a,b)	21.6 ± 4 (a,c)	12.8 ± 5.5 (c)	17.1 ± 4.8 (b)	(a,b,c) < 0.01
Fullness before lunch		28 ± 8.4 (a,b)	18.8 ± 2.5	12.5 ± 4.2 (a)	11.8 ± 4.1 (b)	(a,b) < 0.01
Fullness after lunch		81 ± 5.4 (a)	59.1 ± 19.6 (a,b)	77 ± 5.6 (b)	77 ± 5.6 (a)	(a,b) < 0.05
Prospective consumption before lunch		59 ± 5.5 (a,b,c)	78.3 ± 4 (a)	71.4 ± 3.7 (b)	75.7 ± 4.5 (c)	(a,c) < 0.001 (b) < 0.01
Prospective consumption after lunch		6 ± 2.2 (a,b,c)	25 ± 5.4 (a,b,d)	12.3 ± 2.7 (b,c)	13.5 ± 4.7 (c,d)	(a-b) < 0.001 (d) < 0.01
Palatability		60 ± 7 (a,b,c)	75 ± 5.4 (a)	71.4 ± 4.7 (b)	74.3 ± 5.3 (c)	(a-c) < 0.01

*VAS, Visual Analog Scale is measured in a horizontal scale, maximum value 100. EBD, eating behavior disorder. PD, peritoneal dialysis. Letters represent the statistical differences (read in horizontal) Mann–Whitney U test.*

### Insulin Resistance Markers

Supplementary Figures S1A–E show the changes in glucose, insulin, glucagon, C-peptide and GIP after Fresubin^TM^ intake. PD patients showed higher baseline levels of these markers and “lazy” curves (especially in obese and anorexics) than controls.

### Anorexigenic Peptide Release After Food Intake

**FIGURE 1:**
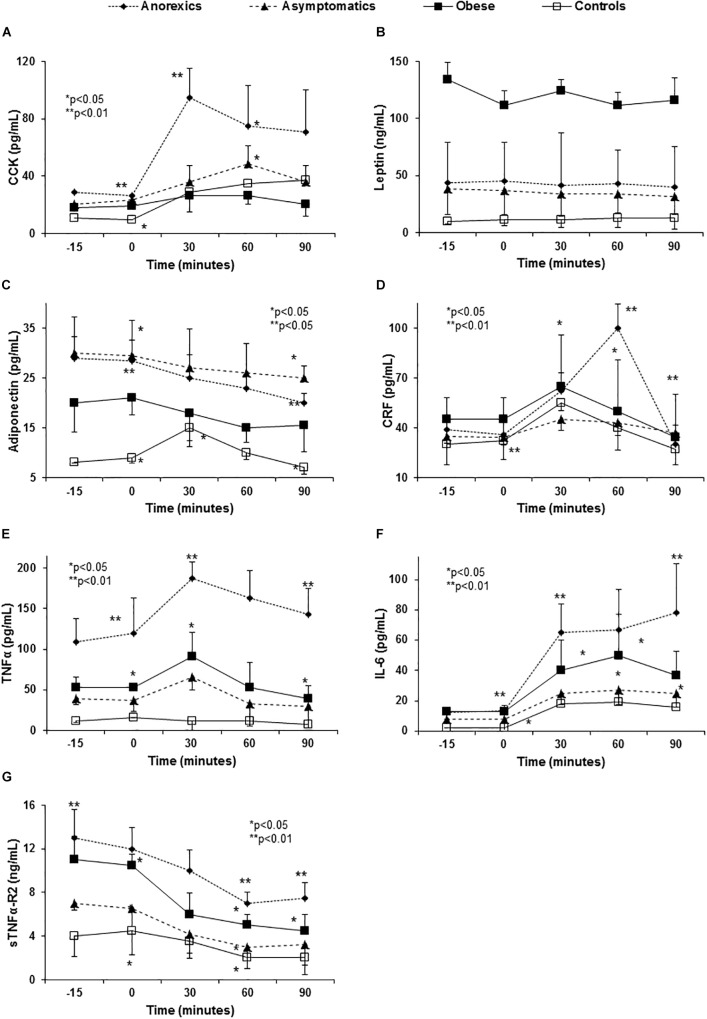
Abnormal anorexigen peptide and pro-inflammatory cytokine release in PD patients with eating behavior disorders. Panel **(A)** shows the CCK release after food consumption. Anorexic patients show a very important increase 30 min after Fresubin^TM^ intake that fell slightly at 60 and 90 min without reaching the basal CCK values. This peak explains the poor appetite (VAS) and the early satiety showed by these patients after food intake (Tables 1–3). The remaining groups had flat curves, except for the controls that maintained the peak values at 30 and 60 min. Obese patients did not show differences along of the curve. *P* < 0.0001 global statistical difference between the groups (three factor ANOVA test). Panel **(B)** shows flat curves of leptin without any modification over time. All PD patients had high basal leptin plasma levels especially the obese group. *P* < 0.01 (three factor ANOVA test). Panel **(C)** shows high adiponectin levels in PD patients, especially in the anorexic and asymptomatic patients when compared with the controls. All PD groups show a progressive and significant decrease over time and the control subjects show a peak at 30 min that fell at 90 min. Panel **(D)** shows the changes in plasma CRF levels after food intake. In normal conditions, CRF levels peak at 30 min and then decrease to values in the normal range after 90 min (controls) *p* < 0.001 (three factor ANOVA test). However, anorexic patients show an important elevation at 60 min possibly perpetuating the lack of appetite started by the CCK peak at 30 min **(A)** and the high basal adiponectin levels **(C)**. *P* < 0.01 (three factor ANOVA test). Panel **(E)** shows the changes in TNF-α plasma levels after standard food intake. Anorexic patients show the highest basal plasma levels and they increase to nearly 200 pg/mL at 30 min, returning to the basal levels after 90 min. Obese and asymptomatic patients have parallel curves with peaks at 30 min and returning to basal levels after 60 min. *P* < 0.01 (three factor ANOVA test). Panel **(F)** shows the changes in IL-6 and the important peak developed by the anorexics patients at 30 min that continued to rise slowly to a maximum after 90 min. In the remaining groups IL-6 peaked at 30 min but it decreased at 60 and 90 min. Panel **(G)** shows the changes in sTNFα-R2 for which the anorexic patients again had the highest values that decrease after eating, while maintaining the highest values over the entire time of the curve. However, after its levels fall at 60 and 90 min, they remained constant. Obese and asymptomatic patients and the controls showed parallel falls. *P* < 0.001 (three factor ANOVA test).

The anorexic patients had high basal and elevated plasma CCK levels 30 min after Fresubin^TM^ intake (Figure 1A), in parallel with the peak rise in glucose and insulin (Supplementary Figures S1A,B). The CCK increment in obese patients was clearly weaker and retarded. In regard to leptin, obese patients showed highest values and flat curve. No single group showed changes in leptin levels (Figure 1B) after food stimulus. In relation to adiponectin, obese patients had the lowest baseline levels from PD groups. All groups showed a postprandial decrease without peaks. On the contrary, the control group showed an important peak at 30 min that declined at 60 and 90 min (Figure 1C).

Corticotropin-releasing factor, other ARP that increased after eating, showed the highest basal levels in obese, with a peak at 30 min and a mild decrease after 90 min. By contrast, the CRF levels in anorexic patients peaked at 60 min with an important decrease after 90 min reaching similar values to the remaining groups (Figure 1D).

### Orexigen Peptide Release After Food Intake

**FIGURE 2:**
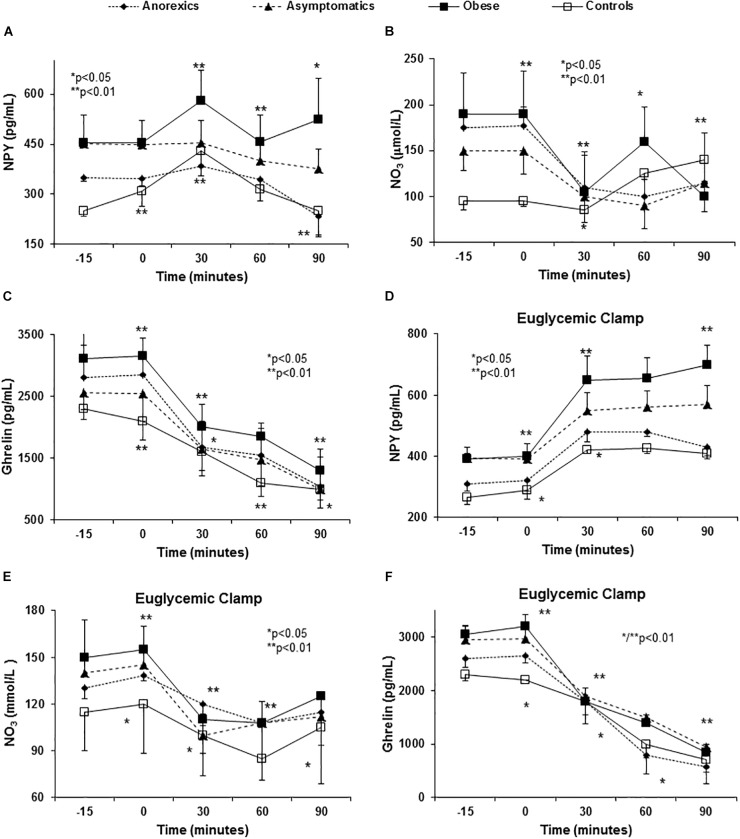
Abnormal orexigenic peptide release (spontaneous and after euglycemic clamp) in PD patients with eating behavior disorders. All PD patients have higher NPY plasma levels (the most orexigenic peptide known) than controls **(A)**. *P* < 0.01 (three factor ANOVA test). In the control group, NPY levels peaked at 30 min followed by a decrease at 60 and 90 min to values below the baseline. Importantly, obese patients show a peak (the highest) at 30 min with a mild fall at 60 min and a rebound at 90 min. The appetite desire shows important correlation with these peaks (Table 2). Asymptomatic and anorexic patients show almost “flat curves.” With regards NO (represented by NO_3_), we found important baseline differences between PD patients and controls. After food intake, all patients show an important fall in plasma NO_3_ levels at 30 min, except asymptomatic patients that show this fall at 60 min with a rebound at 90 min. Obese patients show an early fall, rebound at 60 min and a second fall at 90 min. Anorexic patients had a similar curve as the rest but they did not show a second fall **(B)**. *P* < 0.01 (three factor ANOVA test). The basal ghrelin plasma levels were higher in obese and lower in anorexic patients and an important fall occurs in all patients that is most pronounced in the anorexic patients **(C)**. *P* < 0.01 (three factor ANOVA test). Variance multi-factor analysis (ANOVA). Euglycemic clamp reproduced the abnormalities in orexigenic peptide release found in PD patients. After glucose and insulin infusion, the change in NPY in obese patients shows an important peak at 30 min that was maintained during the study **(D)**. *P* < 0.001 (three factor ANOVA test). Controls and the anorexic patients had lower values than the baseline with a virtually flat curve. The basal plasma NO_3_ levels were higher in the PD patients than in the controls, showing a strong fall to reach similar values as in the controls at 30 min **(E)**. *P* < 0.01 (three factor ANOVA test). The plasma NO_3_ levels were very similar between the groups at later times. With regards ghrelin, the obese patients have higher plasma levels and the anorexic patients the lowest. There was a decrease in NO_3_ after glucose and insulin infusion that followed a very similar pattern in all groups. *P* < 0.01 (three factor ANOVA test). *P* < 0.01 (three factor ANOVA test). The plasma NO_3_ levels were very similar between the groups at later times. There was a decrease in NO_3_ after glucose and insulin infusion that followed a very similar pattern in all groups. With regards ghrelin **(F)** the obese patients have higher plasma levels and the anorexic patients the lowest. *P* < 0.01 (three factor ANOVA test).

Anorexic patients had lower basal levels of NPY and there was a significant decrease in NPY 90 min after eating (Figure 2A–C). The obese patients had higher basal NPY levels and they experienced a significant increase 30 min after eating and remaining stable for up to 90 min (Figure 2A).

Postprandial changes in NO_3_ plasma values were evident and PD patients showed higher basal levels than the controls (Figure 2B). All groups showed an important decrease at 30 min after eating and except in obese patients in whom maximal levels were evident after 60 min, NO_3_ levels peaked after 90 min.

In regard to ghrelin, anorexic patients showed a dramatic fall in ghrelin 30 and 60 min after food consumption (Figure 2C) in comparison with the remaining groups. All groups showed similar values of ghrelin at 90 min after food stimulus.

### Cytokine Release After Food Stimuli

Anorexic patients had higher basal TNF-α and IL-6 levels with important “peaks” 30 and 60 min after food consumption that did not fall after 90 min (Figure 1E,F). The curves from obese and asymptomatic patients displayed intermediate values between those of anorexic and control patients. The values for soluble TNF-α-receptor-2 (sTNFα-R2) were again highest in the anorexic patients and they decreased after eating albeit these patients maintained the highest values at all times (Figure 1G).

### Glucose Clamp (Euglycemic and Hyperglycemic)

We found important differences in insulin sensitivity between PD patients and controls. The obese and anorexic patients showed higher insulin resistance compared with asymptomatic and controls (Supplementary Table S2).

### Induction of Appetite-Related Peptide and Cytokine Release Disorders With Euglycemic Clamp

**FIGURE 3:**
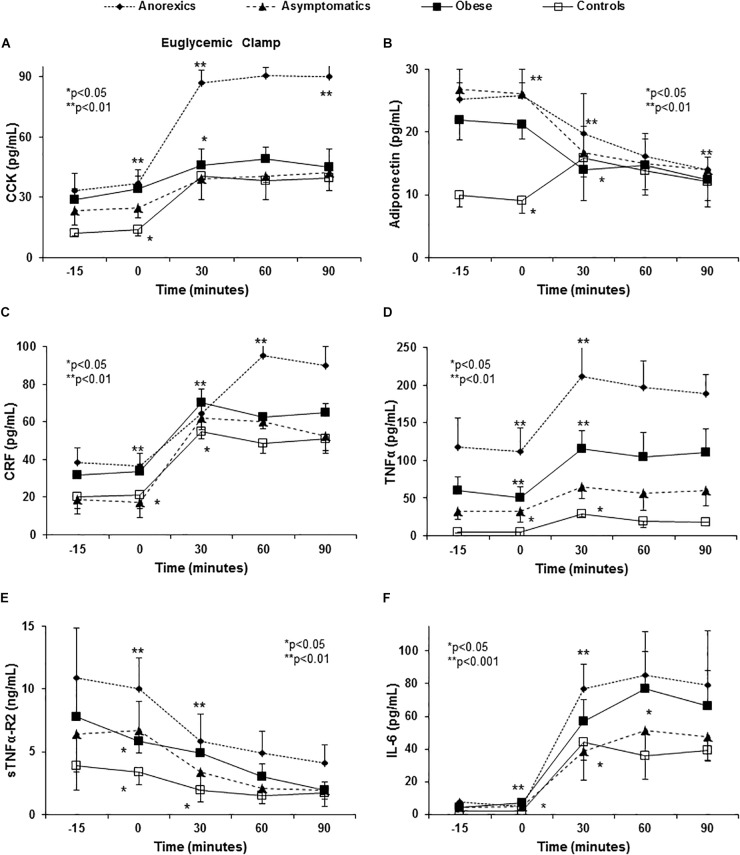
Euglycemic clamp reproduced the abnormalities in anorexigenic peptide pro-and inflammatory cytokine release found in PD patients. A euglycemic clamp permits us to evaluate the effect of insulin and exogenous glucose administration on ARPr, by-passing the gastrointestinal tract. The hyperglycemia and hyperinsulinemia induced are sufficient to abnormally release CCK **(A)**. *P* < 0.001 (three factor ANOVA test). Anorexic patients maintain their CCK peak when compared with the remaining groups. With regards adiponectin, PD patients had higher plasma levels when comparison to the controls **(B)**. *P* < 0.001 (three factor ANOVA test). The obese patients show lower values than the anorexic and asymptomatic patients. All PD patients showed an important fall between 30 and 90 min. By contrast, controls had elevated levels that peaked at 30 min and then fell at 60 and 90 min. Another important anorexigen substance, CRF, also shows important changes and in anorexic patients high levels were reached at 60 min that were maintained until 90 min. The remaining groups show intermediate peaks **(C)**. *P* < 0.01 (three factor ANOVA test). The changes TNF-α, IL-6, and sTNFα-R2 after glucose and insulin infusion are shown in **(D–F)**. Euglycemic clamp reproduced the different curves from anorexic, obese and asymptomatic patients and controls found after eating. However, the ends of the curves (60 and 90 min) were flat, possibly due to the stable and high insulin and glucose levels maintained by the euglycemic clamp. *P* < 0.001 (three factor ANOVA test).

The constant administration of insulin to maintain stable levels of glucose in the euglycemic clamp permitted us to evaluate the influence of exogenous insulin and glucose administration on ARPr, bypassing the GIT. Both stimuli (glucose and insulin) were necessary and crucial to induce ARPr reproducing the abnormal patterns found in anorexic patients (peaks of CCK and CRF, Figure 3A,C), cytokines (peaks of TNF-α, sTNFα-R2, and IL-6) after eating in anorexic patients. Importantly, TNF-α and sTNFα-R2 secretion was consistently higher in anorexic patients (Figure 3D–F), suggesting that TNF-α was not able to auto-regulate its fat receptor (sTNFα-R2) and release. Moreover, in anorexics CCK and CRF maintained their elevated plasma levels at the end of the curves. Similarly, obese patients showed NPY peak with orexigenic action which had not declined after 90 min of follow-up (Figure 2D). NO_3_ and ghrelin showed a dramatic decline at 60 and 90 min (Figure 2E,F) reproducing the similar patterns showed in anorexic and obese patients after food stimulus, except by the last part of the curves.

### Relationship Between Cytokines, ARPr, and Insulin Resistance Marker

Given the known influence of circulating pro-inflammatory cytokines on insulin-glucose metabolism and their eventual relation with ARPr, we study their possible association as a framework. In the whole group, we found a positive linear correlation between plasma TNF-α levels and IL-1 (0.75, *p* < 0.005), as well as between IL-1 and GIP (0.46, *p* < 0.05), IL-1 and HOMA-IR (0.43, *p* < 0.05), and TNF-α and HOMA-IR (0.5, *p* < 0.05). In obese patients, there was a positive linear correlation between TNF-α and leptin (0.56, *p* < 0.05) and there appeared to be a linear correlation with *Ad* although this was not statistically significant (-0.43, *p* < 0.07, NS). These results suggest that there may be a feedback that would perpetuate a metabolic framework that would influence the feeding behavior.

### The Relationship Between ARPr and Cytokines May Promote Uremic Anorexia

In anorexic patients, there was a positive linear correlation between basal IL-1 and CCK (0.45, *p* < 0.05), while TNF-α showed a negative correlation with ghrelin (-0.66, *p* < 0.01). Similarly, NPY showed a negative linear correlation with basal IL-1 (-0.52, *p* < 0.05) and TNF-α (-0.51, *p* < 0.05), and a positive relationship with CRF (0.51, *p* < 0.01). Finally, basal leptin levels were negatively correlated with ghrelin (-0.54, *p* < 0.01). After food consumption, NPY was negatively correlated with basal IL-1 after 30 and 90 min (-0.64, *p* < 0.01 and -0.61, *p* < 0.01, respectively).

### The Relationship Between ARPr and Insulin Resistance May Promote High Food Intake in Obese Patients

In this group, we found a positive linear correlation between basal serum leptin and basal BMI (0.6, *p* < 0.01), fat mass (by BIA: 0.67, *p* < 0.01), NO_3_ (0.51, *p* < 0.05), HOMA-IR (0.66, *p* < 0.01), and *Ad* (-0.54, *p* < 0.01). Likewise, NPY was positively correlated with ghrelin (0.45, *p* < 0.05). After food stimuli, the NPY levels after 90 min showed a positive linear relationship with all points of the leptin curve at baseline, 30, 60, 90 min, (0.51, *p* < 0.05; 0.67, *p* < 0.01; 0.67, *p* < 0.01; 0.63, *p* < 0.01, respectively).

### Relationship Between ARPr, Cytokine, and Appetite Desire (VAS)

Appetite desire was positively correlated with NPY and negatively correlated with CCK (basal and post-stimuli, Figure 2A and 1A, respectively, Tables 3, 4) and TNF-α in the whole group, suggesting a cause–effect relationship between these molecules and VAS. In anorexic patients TNF-α and CCK showed an inverse correlation with appetite desire, especially 30 min after eating (Table 3). TNF-α was associated with the sensation of fullness and poor palatability, as well as to poor prospective food consumption 90 min later. In obese patients NPY showed the strongest relationship with VAS, especially 30 min after food intake, and it was associated with high palatability and a stronger desire for prospective consumption 90 min after eating (Table 3).

**Table 3:** Relationship between anorexigens/orexigens and VAS in anorexics PD patients.

VAS	Peptides (#)	CCK-0 min	CCK-30 min	CCK-60 min	CCK-90 min	NPY-0 min	NPY-30 min	NPY-60 min	NPY-90 min	TNF-α^&^
Desire to eat before lunch		-0.34^*^	-0.6^**^	-0.52^**^	-0.4^*^	0.36^*^	0.33^*^	0.38^*^		-0.56^**^
Desire to eat after lunch			-0.43^*^			0.46^*^	0.4^*^			
Hunger before lunch		-0.41^*^	-0.66^**^	-0.47^*^	-0.5^**^			0.34^*^		-0.66^**^
Hunger after lunch			-0.55^**^	-0.52^**^	-0.53^**^					
Fullness after lunch					0.55^**^	-0.46^*^	-0.6^**^		0.51^**^	0.52^**^
Prospective consumption before lunch					-0.48^*^	0.48^*^	0.34^*^		0.47^*^	-0.48^*^
Prospective consumption after lunch				-0.4^*^				0.56^**^		
Palatability		-0.5^*^	-0.53^**^		-0.75^**^		0.42^*^		0.42^*^	-0.6^**^
Hunger 2 h before lunch			-0.38^*^	-0.4^*^	-0.6^**^			0.34^*^		-0.5^**^
Satiety 2 h after lunch			0.53^**^		0.42^*^					

*VAS, Visual Analog Scale. PD, peritoneal dialysis. #, means the time after the food stimulus; &, baseline. ^∗^p < 0.05, ^∗∗^p < 0.01.*

**Table 4:** Relationship between anorexigens/orexigens and VAS in obese PD patients.

VAS	Peptides (#)	CCK-0 min	CCK-30 min	CCK-60 min	CCK-90 min	NPY-0 min	NPY-30 min	NPY-60 min	NPY-90 min	TNF-α^&^
Desire to eat before lunch			-0.41^**^		-0.38^*^	0.5^**^	0.58^**^		0.4^*^	
Desire to eat after lunch		-0.38^*^		-0.38^*^	-0.48^*^		0.54^**^			
Hunger before lunch		-0.5^*^	-0.36^*^	-0.58^**^	-0.35^*^	0.4^*^	0.38^*^	0.55^**^		-0.31^*^
Hunger after lunch			-0.38^*^	-0.39^*^	-0.33^*^	0.33^*^	0.54^**^		0.5^**^	
Fullness after lunch		0.43^*^				-0.34^*^	-0.34^*^			
Prospective consumption before lunch						0.57^**^	0.6^**^	0.37^*^	0.53^**^	-0.33^*^
Prospective consumption after lunch				-0.37^*^			0.53^**^	0.51^*^	0.53^**^	
Palatability		-0.46^*^		-0.56^**^			0.41^*^		0.63^**^	-0.37^*^
Hunger 2 h before lunch					-0.35^*^		0.51^**^	0.56^**^	0.7^**^	
Satiety 2 h after lunch				0.35^*^					-0.44^*^	

*VAS, **V**isual Analog Scale. #, means the time after the food stimulus; &, baseline. Linear correlations: ^∗^p < 0.05, ^∗∗^p < 0.01.*

### Appetite-Related Gene Expression in Abdominal Fat Tissue

**FIGURE 4:**
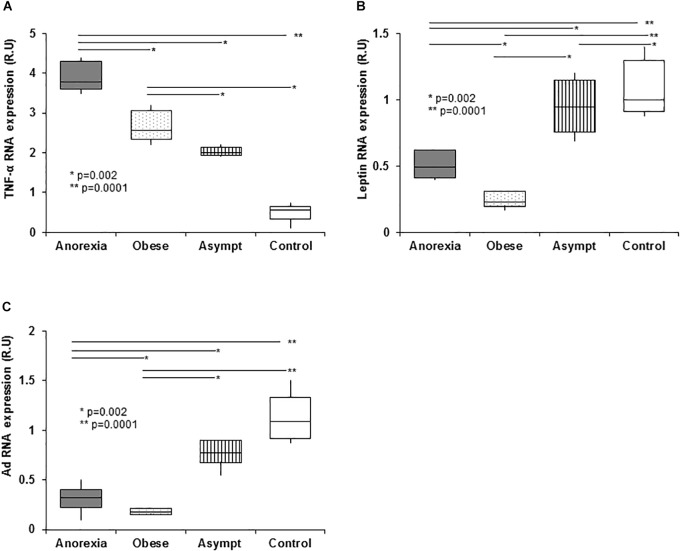
Expression of genes related to eating behavior disorders in PD patients in abdominal subcutaneous fat (qPCR). PD patients show higher fat tissue TNF-α expression than controls. The highest TNF-α expression was in the anorexic patients, with obese and asymptomatic patients showing intermediate expression. The high TNF-α plasma levels present in anorexic patients did not inhibit the TNF-α expression in fat suggesting that in uremia, fat tissue is an important source of pro-inflammatory cytokines **(A)**. With regards leptin expression in fat tissue, the obese patients show an important downregulation of leptin gene expression in fat when compared to the remaining groups. This down expression may be associated with a negative feedback loop induced by the high plasma TNF-α levels. Anorexic and asymptomatic patients express intermediate levels of leptin between obese patients and controls **(B)**. Panel **(C)** shows the adiponectin expression, which was lower in the fat from PD patients than in controls. As expected, obese patients expressed adiponectin the weakest and again, anorexic and asymptomatic patients had expression levels intermediate to those of obese patients and controls. Box plots show the 25th and 75th percentiles, median, minimum and maximum values of five independent experiments. The symbols represent the statistical differences between the groups (ANOVA one-way and Mann–Whitney rank sum *U* test).

The TNF-α gene was over-expressed in all uremic patients, and anorexic patients showed higher mRNA expression than other groups (Figure 4A). Importantly, TNF-α gene expression was positively correlated with TNF-α plasma levels (0.66, *p* < 0.05). Obese patients had the lowest fat leptin expression, which was negatively correlated with plasma leptin levels (-0.54, *p* < 0.0.5), indicating a negative feedback that down-regulated the expression of this gene (Figure 4B). All patients showed lower global Ad fat expression than controls, and the lowest expression was evident in obese patients (Figure 4C).

### Relationship Between Fat Appetite-Related Gene Expression and Insulin Resistance

There was a positive linear correlation between plasma insulin (Supplementary Figure S1B) and fat TNF-α expression (0.55, *p* < 0.01), as well as a negative relationship between plasma insulin and fat leptin expression (0.34, *p* < 0.05) or fat Ad expression (0.44, *p* < 0.05) (Figure 4A–C). Similarly, HOMA was positively related with fat TNF-α expression (0.64, *p* < 0.01) and it was negatively related to fat leptin expression (0.5, *p* < 0.01).

**Table 5:** Relationship between fat gene expression and appetite desire (VAS).

	Anorexics			Obese patients		
VAS/gene expression	TNF-α	Leptin	Adiponectin	TNF-α	Leptin	Adiponectin
Desire to eat before lunch	-0.66^**^	-0.55^**^	-0.54^**^		0.37^*^	
Desire to eat after lunch	-0.43^*^				0.45^*^	
Hunger before lunch	-0.4^*^	-0.55^*^	-0.34^*^			-0.4^*^
Hunger after lunch						
Fullness after lunch	0.7^**^			0.37^*^		
Prospective consumption before lunch		-0.6^**^			0.56^**^	
Prospective consumption after lunch		-0.46^*^	-0.37^*^			-0.5^*^
Palatability	-0.5^**^					
Hunger 2 h before lunch	-0.41^*^			-0.35^*^	0.44^*^	
Satiety 2 h after lunch		0.5^*^				

*VAS, Visual Analog Scale. Linear correlations: ^∗^p < 0.05, ^∗∗^p < 0.01.*

### Relationship Between Fat Appetite-Related Gene Expression and Appetite Desire (VAS)

Table 5 shows the relationships between TNF-α, leptin and Ad gene expression, and appetite, measured by VAS, in anorexic and obese patients. The participation of TNF-α and its association with VAS was predominant in anorexic patients.

## Discussion

There are three important findings arising from this study:

(1) We have identified well-defined patterns of EBD in dialysis patients, defined by the VAS and nutritional markers (Table 1 and Supplementary Table S2).
(2) The presence of ARPr disorders associated with insulin resistance and systemic inflammation (Tables 3, 4 and Figure 1–4).
(3) The close relationship between EBD, abnormal ARP, and cytokines release (Tables 3, 4) and cytokines fat-tissue gene expression (Table 5 and Figure 4).

### Characterization of Appetite Disorders in PD Patients

Visual Analog Scale has been used to measure eating motivation and validated in PD, HD, and kidney transplant patients (Hylander et al., 1997). Employing this scale, we demonstrate that patients suffering anorexia showed the minimal score of hunger, lower palatability and eating desire, in conjunction with a sensation of greater fullness, contrasting with the features found in obese patients. Importantly, our patients showed a dietary preference for carbohydrates.

### Uremic Insulin Resistance

As it has been demonstrated by others (Zoccali et al., 2005; Kalra et al., 2008), we found different grades of insulin resistance in our PD patients that were related with different EBD.

### Baseline Results

Our PD patients showed high spontaneous baseline plasma levels of anorexigenic peptides and cytokines, including C-peptide and GIP, which have an anorexigenic effect mediated by insulin-glucose metabolism. Importantly, anorexic patients have the highest plasma levels of anorexigens including TNF-α and low NPY levels (orexigen), as we previously described (Aguilera et al., 1998, 2001). IL-1 and IL-6 are another cytokines associated with loss of appetite (McCarthy et al., 1995). According to our results, patients with anorexia have the lowest RRF and the highest cytokine concentration, suggesting that RRF is a more important determinant of appetite through this *via* than dialysis dose.

### Results Following Food Intake

We clearly identified distinct alterations in ARPr pattern in studied groups after food intake. Given these results, we explored the influence of exogenous insulin and glucose administration, together or separately (hyperglycemic and euglycemic clamp), on ARP and cytokine release bypassing the GIT.

### Anorexigen Substances

Cholecystokinin is a potent anorexigen with peripheral and central actions that is implicated in the pathogenesis of *anorexia nervosa*, cancer, senile and alcoholic anorexia (Dupré et al., 1973). In PD patients with anorexia, the plasma levels of CCK were elevated after eating. CCK is also retained in dialysis patients and it is not modified by PD or HD (Peikin, 1989). Previously, we did not find high CCK plasma levels in anorexic patients prior to food stimulus (Aguilera et al., 1998). Here, we identified a “peak” of plasma CCK 30 min after eating (Figure 1A), which may be responsible for the early sensation of fullness. This CCK “peak” was also found in patients with anorexia nervosa and although the exact cause is unknown, this phenomenon is potentially reversible when the nutritional status is recovered (Owyang et al., 1979). Modifications to peripheral insulin activity when nutrition or systemic inflammation improves might explain this phenomenon. By contrast, obese patients showed a delayed CCK “peak” and a similar abnormality may be found in non-uremic patients with bulimia nervosa, where the CCK “peak” is delayed and is 50% lower than those in control (Harty et al., 1991).

Again, the positive linear correlations found between CCK and IL-1, IL-1, and GIP, and GIP and insulin (other anorexigen) may perpetuate the anorexia and the insulin resistance suffered by our patients (Daun and McCarthy, 1993). In fact, a synergic effect between IL-1 and CCK inducing anorexia has been described, while IL-1 and GIP are factors that stimulate insulin release by the pancreas (Geracioti and Liddler, 1988). The euglycemic clamp replayed the same CCK and cytokines release patters (Figure 3A,D–F). These results suggest that insulin metabolism and high grade of systemic inflammation may be key in the induction of anorexia in PD patients.

Leptin is an adipose tissue hormone that modulates appetite and insulin activity in target cells, inducing insulin resistance (Klein et al., 1996). Although, we did not find changes in leptin levels, in non-renal population plasma leptin appears to increase 4 h after food intake (Klein et al., 1996). Unfortunately, we have no data from this time interval.

Adiponectin, in sharp contrast to leptin, plasma *Ad* levels are negatively correlated with body fat, decreasing with obesity and increasing with weight loss (Liu et al., 2003; Coll et al., 2007). Moreover, hyperadiponectinemia can reduce the food intake in rats (Coope et al., 2008). Here, we found high plasma *Ad* levels in PD patients, although, the lowest values were registered in obese patients as described elsewhere in non-renal obese (Coll et al., 2007). In normal conditions, *Ad* levels increase 30 min after food intake and decrease over the following 60 and 90 min. In all our patients the decrease was delayed, the obese patients maintaining the lowest values. It is generally accepted that *Ad* is negatively related to insulin levels due to its anti-insulin resistance effects (Isganaitis and Lustig, 2005). However, in uremia, the negative feedback between insulin secretion and *Ad* may be partially broken since adiponectin is retained (Díez et al., 2005). Indeed, the exogenous administration of insulin and glucose in the euglycemic clamp strongly regulated *Ad* in all studied groups (Figure 3B). Therefore, the differences in *Ad* release in these patients appear to be mediated by insulin resistance contributing indirectly to EBD in PD patients.

Corticotropin-releasing factor is produced by hypothalamic neurons that exert a central and peripheral anorexigenic effect, inhibiting that of NPY (Kalra et al., 2008). While there were no basal differences between PD patients, 60 min after eating anorexic patients displayed an important peak of CRF that was correlated with VAS. In addition to insulin, one of the most important regulators of CRF activity is plasma cortisol, therefore impaired cortisol suppression may explain EBD in uremia (Deshmukh et al., 2005). Unfortunately, we did not study the circadian variations in plasma cortisol and ACTH. In accordance with our findings, an important disruption in insulin release that may be associated to disorders in CRF secretion and *vice-versa* has already been observed in patients suffering chronic renal failure (Feneberg et al., 2002). Again euglycemic clamp reproduced the CRF release patters in all groups suggesting a deep dependence of insulin and glucose levels (Figure 3C).

### Orexigenic Substances

In normal condition a “peak” of NPY appeared 15–30 min after food intake and may be responsible for hunger persistence, possibly augmenting the sensation of gastric emptiness (Weisman et al., 1998). This explains the popular idea that small snacks before the main meal stimulate appetite. Importantly, this NPY “peak” was absent in our patients with uremic anorexia (Figure 2A), who conversely showed a significant decrease in NPY 90 min after eating that fell below the basal values, explaining the early and late lack of appetite seen in anorexics. NPY “peaks” were evident 30 and 90 min after eating in obese patients, which could explain the repetitive and greater amount of food ingested. This post-prandial NPY “peak” rebound at 90 min has been described previously in bulimic patients (Rosenbaum et al., 1997). Moreover, our obese group showed relatively high NPY levels, although within the normal range, which could be associated with high appetite desire. Recent studies suggest the existence of disorders in hunger-hypothalamic receptor sensibility in the non-uremic obese population (Rosenbaum et al., 1997; Weisman et al., 1998). NPY and leptin levels are significantly higher in Zuker (*Ob/Ob*) rats, inducing repetitive and compulsive food intake due to the failure of leptin to inhibit NPY release (Stricker-Krongrad et al., 1994). In uremic status, hypothalamic receptor disorders have been poorly studied, although the parallels in the glucose, insulin and NPY curves and their complex relationship may underlie the excessive and sustained NPY release. Obesity and uremia status may be the maximal expression of this disorder since both are associated with the hyperinsulinemia and insulin resistance that might perpetuate the abnormal NPY release. The euglycemic clamp results support the role of insulin metabolism on NPY release (Figure 2D).

But not only carbohydrate intolerance appears to affect appetite in uremia. Cytokines also may be affected by insulin metabolism. We recently demonstrated an inverse relationship between TNF-α and NPY in PD patients, and we speculated that TNF-α may inhibit its orexigenic effect (Xu et al., 1988; Aguilera et al., 1998). We also found a negative linear correlation between NPY and IL-1, supporting the MIA hypothesis (Aguilera et al., 1998). Together, ARPr, TNF-α, IL-6, and s-TNFα-R2 showed important modifications after food stimulus. This parallelism invites to think that pro-inflammatory cytokines may synergize and perpetuate EBD as anorexia in our patients.

Nitric oxide is another important appetite stimulator (Xu et al., 1988; Vallance et al., 1992; Squadrito et al., 1994) and experimentally, a decrease in NO production inhibits appetite (Squadrito et al., 1994). A potent NO-synthase inhibitor accumulated in uremic patients (Vallance et al., 1992) and as a consequence, one might expect dialysis patients to have lower plasma NO_3_ values than controls. However, it did not occur. This apparent contradiction may be explained by the uremic retention of inactive forms of NO, represented by NO_3_, by the NO relationship with the pool of arginine (its substrate), ornithine, pro-inflammatory cytokines capable of inhibiting NO synthase, endothelin-1 and insulin metabolism (Yan et al., 2008). NO stimulates the insulin release and the equilibrium between NO and endothelin-1 with a predominium of endothelin-1 inducing insulin resistance (Yan et al., 2008). Moreover, high plasma levels of endothelin-1 have been reported in uremics (Roccatello et al., 1997).

On the other hand, modification of NO after food intake and after the euglycemic clamp highlights the role of insulin and uremic insulin resistance on hunger-satiety control (Figure 2E). Moreover, the high TNF-α plasma levels shown by anorexic patients may explain the dramatic fall in NO_3_ after food stimuli due to decreased NO synthesis perpetuating the early anorexia (Yan et al., 2008).

Ghrelin is another orexigen GIT hormone that is secreted in response to stomach empty. It decreases after food intake, constituting a peripheral negative feedback and increase after weight loss, in fasting condition and insulin induced hypoglycemia (Theander-Carrillo et al., 2006). Ghrelin plasma levels in uremia are generally elevated, although anorexic patients show relatively lower values (Aguilera et al., 2004a). We confirmed our previous findings, that PD patients maintain higher ghrelin plasma levels than controls (Figure 2C). Moreover, we confirmed the strong inverse relationship between ghrelin levels and glucose or insulin release after food intake, and that after euglycemic clamping ghrelin maintained its levels in parallel with the insulin and glucose levels (Figure 2F).

Not only insulin resistance and inflammation participate in appetite regulation, but the expression of genes in abdominal fat associated with insulin metabolism can also regulate appetite. Fat TNF-α over-expression (Figure 4A) is not inhibited by its high plasma levels and in fact, the levels of sTNFα-R2 are maintained, indicating that fat tissue is an important source of pro-inflammatory cytokines in uremia. By contrast, obese subjects have the lowest leptin and *Ad* fat expression (Figure 4B,C) indicating their down-regulation in fat, possibly due to negative feedback through their high plasma, as described by elsewhere (Nordfors et al., 1998).

Our results permit to explain the different eating behaviors in PD patients according to different degrees of insulin resistance and systemic inflammation. The euglycemic clamp exactly reproduces the insulin sensitivity and abnormal ARP and cytokines release patterns in anorexics and obese, indicating that glucose-insulin metabolism definitely triggers these appetite disorders.

Finally, other abnormalities as sex hormones, brain neurotransmitters or receptors acquired disorders in uremia (i.e., MC4-r), may contribute to the diversity of EBD in this population (Xu et al., 1988; Cheung et al., 2005; Carrero et al., 2008).

## Conclusion

In PD patients, EBD are modulated by an abnormal baseline levels of ARP and cytokines, which are abnormally released after food intake and highly dependent of insulin metabolism. The renal ARP retention, the excess of pro-inflammatory cytokines and uremic carbohydrate intolerance, with predominance of inflammation in anorexics or hyperinsulinemia in obese patients, may explain the tendency to develop one or other EBD.

## Ethics Statement

The present study adjusts to the Declaration of Helsinki and was approved by the Ethics Committee of the Hospital Universitario la Princesa, Madrid, Spain. Informed written consent was obtained from all the patients.

## Author Contributions

All authors listed have made a substantial, direct and intellectual contribution equally to the work, and approved it for publication.

## Conflict of Interest Statement

The authors declare that the research was conducted in the absence of any commercial or financial relationships that could be construed as a potential conflict of interest.

## Supplementary Material

FIGURE S1 Insulin resistance markers release in PD patients with eating behavior disorders. Panel **(A)** shows the glucose release after standard food intake (Fresubin^TM^). PD patients, especially obese patients, show an important and sustained elevation of glucose that reaches values between 150 and 165 mg/dL. In the control group glucose peaks at 30 min, decreasing to normal values at 60 min and then declining at later periods (90 min). *P* < 0.001 (three factor ANOVA test). Panel **(B)** shows a similar pattern of insulin release as glucose. In obese the insulin curve was highest and maintained around 150 μU/mL a long the time (90 min). All PD patients showed “lazy curves” with insulin levels between 50 and 90 μU/mL, whereas controls show a final decrease in insulin at 90 min. *P* < 0.001 (three factor ANOVA test). Panel **(C)** shows the glucagon plasma levels in the different groups. Controls show a mild elevation at 60 min with a significant decrease at 90 min. In PD patients the levels remain constant (“flat curves”) except for the peak detected in obese patients at 30 min. *P* < 0.01 (three factor ANOVA test). Panel **(D)** shows the C-peptide curves and again, obese patients have the highest values in the curve. The controls show a peak at 60 min with a significant decrease at 90 min. *P* < 0.001 (three factor ANOVA test). Panel **(E)** shows the GIP levels in the groups studied. PD patients display an important elevation at 30 and 60 min (anorexics) with no decrease at later periods, whereas the lowest values were in the controls maintaining the peak at 60 and 90 min. Obese patients showed an important and sustained elevation of glucose that reaches values of 150–165 mg/dL. Control group showed a glucose peaks at 30 min, decreasing to normal values at 60 min and then declining at 90 min. In whole PD patients, a similar pattern of insulin release and glucose was found “lazy curves.” Obese showed the highest insulin values with fat curves at later periods, whereas controls show a final decline in insulin curve at 90 min. In regard to glucagon, patients showed elevated baseline levels than controls, especially in obese and anorexics with a small peak at 30 and 60 min, respectively. Controls show a mild elevation at 60 min with a significant decrease at 90 min. The C-peptide a pancreatic insulin reserve marker, showed the highest values in obese. Controls show a peak at 60 min with a significant decrease at 90 min. Figure 1E shows the changes in GIP levels after food stimulus. Anorexic PD patients display an important elevation of GIP at 30 and 60 min which is maintained at later periods, whereas controls showed lowest values with peak at 60 and decreasing at 90 min. *P* < 0.01 (three factor ANOVA test).

TABLE S1 Gene primers.

TABLE S2 Euglycemic (insulin sensitivity) and hyperglycemic clamp study (insulin secretion).
