# Supplementary figures and images for: Abnormalities in Glucose Metabolism, Appetite-Related Peptide Release, and Pro-inflammatory Cytokines Play a Central Role in Appetite Disorders in Peritoneal Dialysis
Source: Front Physiol. 2019 May 28;10:630. doi: 10.3389/fphys.2019.00630 (PMC6547940; doi:10.3389/fphys.2019.00630)

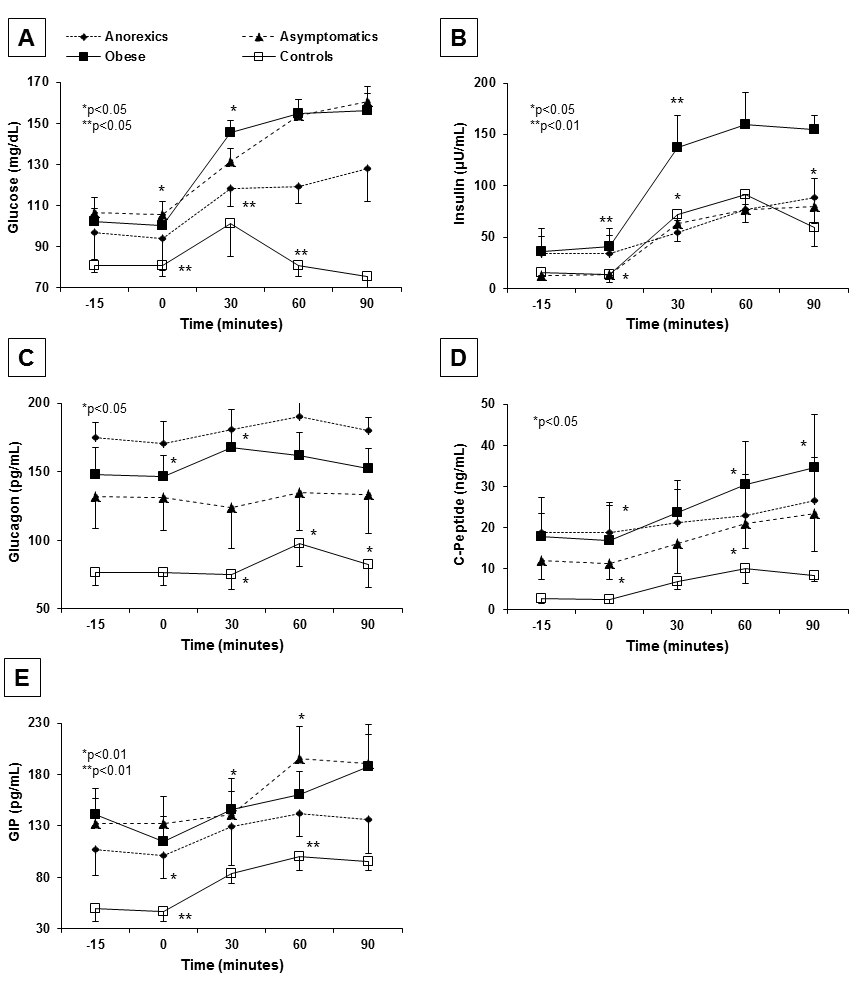

Supplement: FIGURE S1 — Insulin resistance markers release in PD patients with eating behavior disorders. Panel (A) shows the glucose release after standard food intake (FresubinTM). PD patients, especially obese patients, show an important and sustained elevation of glucose that reaches values between 150 and 165 mg/dL. In the control group glucose peaks at 30 min, decreasing to normal values at 60 min and then declining at later periods (90 min). P < 0.001 (three factor ANOVA test). Panel (B) shows a similar pattern of insulin release as glucose. In obese the insulin curve was highest and maintained around 150 μU/mL a long the time (90 min). All PD patients showed “lazy curves” with insulin levels between 50 and 90 μU/mL, whereas controls show a final decrease in insulin at 90 min. P < 0.001 (three factor ANOVA test). Panel (C) shows the glucagon plasma levels in the different groups. Controls show a mild elevation at 60 min with a significant decrease at 90 min. In PD patients the levels remain constant (“flat curves”) except for the peak detected in obese patients at 30 min. P < 0.01 (three factor ANOVA test). Panel (D) shows the C-peptide curves and again, obese patients have the highest values in the curve. The controls show a peak at 60 min with a significant decrease at 90 min. P < 0.001 (three factor ANOVA test). Panel (E) shows the GIP levels in the groups studied. PD patients display an important elevation at 30 and 60 min (anorexics) with no decrease at later periods, whereas the lowest values were in the controls maintaining the peak at 60 and 90 min. Obese patients showed an important and sustained elevation of glucose that reaches values of 150–165 mg/dL. Control group showed a glucose peaks at 30 min, decreasing to normal values at 60 min and then declining at 90 min. In whole PD patients, a similar pattern of insulin release and glucose was found “lazy curves.” Obese showed the highest insulin values with fat curves at later periods, whereas controls show a final decline i [file Image_1.TIF]
