# Supplementary material for: Abnormalities in Glucose Metabolism, Appetite-Related Peptide Release, and Pro-inflammatory Cytokines Play a Central Role in Appetite Disorders in Peritoneal Dialysis
Source: Front Physiol. 2019 May 28;10:630. doi: 10.3389/fphys.2019.00630 (PMC6547940; doi:10.3389/fphys.2019.00630)
Supplement: TABLE S1 — Gene primers. [file Table_1.DOC]

#### Supplementary table I. Gene primers

____________________________________________________________________________

**Forward Reverse**

____________________________________________________________________________

Adiponectin 5’-TGGTGAGAAGGGTGAGAA-3' 5’-AGATCTTGGTAAAGCGAATG-3’

Leptin 5’-TTGGCCCTATCTTTTCTATG-3’ 5’-GCATACTGGTGAGGATCTGT-3'

TNF-α 5`-GAGCACTGAAAGCATGATCC-3’ 5’-GCTGGTTATCTCTCAGCTCCA-3’

GAPDH 5’-TGGTCTCCCTCTGACTTCAAC-3’ 5’-GTGAGGGTCTCTCTCTTCCT-3’

____________________________________________________________________________
