# Supplementary material for: Abnormalities in Glucose Metabolism, Appetite-Related Peptide Release, and Pro-inflammatory Cytokines Play a Central Role in Appetite Disorders in Peritoneal Dialysis
Source: Front Physiol. 2019 May 28;10:630. doi: 10.3389/fphys.2019.00630 (PMC6547940; doi:10.3389/fphys.2019.00630)
Supplement: TABLE S2 — Euglycemic (insulin sensitivity) and hyperglycemic clamp study (insulin secretion). [file Table_2.DOCX]

**Supplementary Table 2. Euglycemic (insulin sensitivity) and Hyperglycemic Clamp Study (insulin secretion).**

| **Euglycemic**  **Clamp** | Anorectic  (PD) | Obese patients (PD) | Asymptomatic patients (PD) | Controls | (p) between groups |
| --- | --- | --- | --- | --- | --- |
| Insulin sensitivity (mg/m^2^/min) | 201 ± 12  (a,b) | 172.6 ± 20.2  (a,c) | 234 ± 17.1  (d) | 346 ± 18.1  (a,b,c) | (a,b,d) <0.05  (c) <0.01 |
| **Hyperglycemic Clamp (µU/mL)** |  |  |  |  |  |
| Baseline  (-15 min) | 30 ± 18  (a,b) | 42 ± 13.7 | 15 ± 4  (a) | 11 ± 2,4  (b) | (a,b ) <0.05 |
| Baseline  (0 min) | 33 ± 22.2  (c,d,φ,∂, Ψ) | 39.8 ± 10.2  (e,π) | 14.1 ± 5  (d,α,ω,*) | 10.2 ± 3.4  (c,e,χ,^+^) | (c,d,e) <0.05 |
| 30 min | 61.2 ± 10  (f,φ) | 140.1 ± 36  (f,g,π) | 51.3 ± 12.4  (f,α) | 54.2 ± 5.4  (g,χ) | (f,g) <0.05 |
| 60 min | 79 ± 21.6  (h,∂) | 160.4 ± 21.4  (h,i,π) | 56.9 ± 14.5  (h,ω) | 73 ± 15.3  (i,^+^) | (h,i) <0.05 |
| 90 min | 88 ± 15.6  (j,Ψ) | 175 ± 26.5  (j,k,l,π) | 73.5 ± 14  (k,*) | 45.3 ± 9.8  (l) | (j-l) <0.05 |
| post prandial changes (p) | (φ,∂,Ψ) <0.05 | (π) <0.05 | (α,ω,*) <0.05 | (χ,^+^) <0.01 |  |

Letters represent statistic differences between the groups (read in horizontal)

Symbols represent statistic differences along the time (read in vertical)
